# Effect of Icariin on Tibial Dyschondroplasia Incidence and Tibial Characteristics by Regulating P2RX7 in Chickens

**DOI:** 10.1155/2018/6796271

**Published:** 2018-03-20

**Authors:** Hui Zhang, Khalid Mehmood, Xiong Jiang, Wangyuan Yao, Mujahid Iqbal, Kun Li, Xiaole Tong, Lei Wang, Meng Wang, Lihong Zhang, Fazul Nabi, Mujeeb Ur Rehman, Jiakui Li

**Affiliations:** ^1^College of Veterinary Medicine, Huazhong Agricultural University, Wuhan 430070, China; ^2^University College of Veterinary & Animal Sciences, Islamia University of Bahawalpur, Bahawalpur 63100, Pakistan; ^3^College of Animals Husbandry and Veterinary Medicine, Tibet Agricultural and Animal Husbandry University, Linzhi, Tibet 860000, China

## Abstract

Tibial dyschondroplasia (TD) is a disease of rapid growing chickens that occurs in many avian species; it is characterized by nonvascular and nonmineralized growth plates, along with tibia bone deformation and lameness. Icariin is widely used to treat bone diseases in humans, but no report is available regarding the effectiveness of icariin against avian TD. Therefore, this study was designed to determine its effect against TD. For this purpose, a total of 180 broiler chicks were distributed into three groups including control, TD, and icariin group. Control group was given a standard normal diet, while TD and icariin groups received normal standard diet containing 50 mg/kg thiram to induce TD from days 3 to 7 after hatch. After the induction of TD, the chicks of icariin group were fed with standard normal diet by adding 10 mg/kg icariin in water. Then morphological and production parameters analysis of tibial bone indicators, physiological index changes, and gene expression were examined. The results showed that icariin administration not only decreased the mortality but also mitigated the lameness and promoted the angiogenesis, which diminished the TD lesion and significantly increased the expression of P2RX7 (*P* < 0.05) in TD affected thiram induced chicks. In conclusion, present findings suggest that icariin has a significant role in promoting the recovery of chicken growth plates affected by TD via regulating the P2RX7. Our findings reveal a new target for clinical treatment and prevention of TD in broiler chickens.

## 1. Introduction

Tibial dyschondroplasia (TD) is a tibiotarsal bone disorder which is linked with rapidly growing avian species, especially broilers [1] and turkeys [2]. It is characterized by dull white nonvascularized and nonmineralized lesion from the epiphyseal growth plate (GP) to the proximal tibiotarsal bone. TD is attributed to abnormal differentiation of chondrocytes which ultimately leads to overt locomotion problem [3–5]. It was first described in turkeys in 1978, and a recent statistics showed that TD was almost 30% of the bone disorders in chickens and turkeys that caused enormous economic losses and animal welfare problem [6]. TD is an important disease of poultry industry, which leads to leg problem by disturbing the proximal growth plate of the tibia bone [7]. However, there is no systematic study to describe the mechanisms leading to TD development. Previous research indicates that the epiphyseal growth plate chondrocytes show continuous cell proliferation, hypertrophy, remodeling, and cell removal in early phase of the growth [8]. The development of GP is constitutive of different regions, germinal, proliferating, prehypertrophic, and hypertrophic zone, which accommodate chondrocytes with discrete morphology along with deeply penetrating metaphysical trabecular bone and blood vessels [9, 10].

Although the exact pathogenesis of TD is still not clear, many researchers have speculated the possible pathogenesis and treatment of this disorder [10–13]. The recent studies showed that the nutrition, selective breeding, and rearing environment affect the incidence of TD [3, 12, 14]. However, limited studies have shown the systematic approach to investigate the changing rule of bone remodeling in TD chickens.

The mechanism of bone remodeling is to maintain the skeleton and replacement of damaged bone [15]. The pathway of bone remodeling is the mechanical stimuli, cellular responses, and mechanotransduction, but previous studies have shown that the nucleotide discharge and successive P2 receptor activation also play an important role in this pathway [16–18]. More recently, disruption of gene encoding P2X7 receptor in mice suggested its role in the regulation of bone formation and resorption [18, 19]. The P2X7 nucleotide receptor (P2X7R) is expressed in bone and plays important role in bone metabolism [20]. Mice with a null mutation of the P2X7R were shown to have a reduced total bone mineral content, reduced periosteal bone formation, and increased trabecular bone resorption [19, 21].

Icariin is a natural flavonoid glycoside isolated from some species of plants belonging to the genus* Epimedium* [22]. Traditionally, it is widely recorded for the treatment of erectile dysfunction in China [22, 23]. However, much attention has been given towards its potential to treat bone problems, cardiovascular, which are demonstrated as a potential osteogenic compound for bone repairmen and bone formation. Most interestingly, it has been reported that bone regeneration was significantly enhanced after the implantation of icariin clinically [24, 25].

Previous reports suggest that icariin has efficient chondroprotective activities [26], and it can prevent degradation of bone in arthritis by reducing chondrocytes destruction [27]. Zhang et al. [28] stated that icariin can induce chondrogenesis by promoting gene expression of chondrocytes and extracellular matrix synthesis. So, we hypothesized that icariin could prevent TD by improving the bone formation and repairing the affected GPs. Thus, the aim of the present study was to investigate the possibility of icariin action against thiram induced TD in chickens via regulating the expression of P2XR7.

## 2. Materials and Methods

### 2.1. Chick's Management and Experimental Plan

A total of 180-day-old broiler chickens (Arbor Acres) of weighing 48 ± 6 g were brought from a commercial hatchery. All groups were offered ad libitum regular diet as suggested by the National Research Council [29]. The experiments were conducted after the approval of Institutional Animal Welfare and Research, Ethics Committee of Huazhong Agricultural University, Wuhan, China.

One hundred and eighty chickens were equally divided into control group, TD group, and icariin group. TD and icariin group were given standard normal along with the addition of thiram 50 mg/kg of feed from day 3 after hatch to induce TD. After induction of TD (on day 8) the icariin group was separated and fed with standard normal diet along with addition of 10 mg/kg/day icariin through drinking water until the end of the experiment. From day 8, the TD group was offered standard normal feed just like control group.

### 2.2. Morphological and Production Parameters Analysis and Sample Collection

The chicken groups were raised for 18 days and the number of morbidity, mortality, and lame birds (walking abilities and the period they were able to remain standing) was recorded in each group on daily basis. During the experimental period, 10 chicks were sacrificed by cervical dislocation randomly on days 7, 10, 14, and 18 from each group. After euthanizing, the tibia bones were measured for morphological examination including length, width, size of tibial GP, and weight by Digital Calipers and electronic balance, respectively. The TD score and severity of TD were determined according to Pines et al. [6] and Simsa et al. [2]. Briefly, TD score 0 = normal growth plate; 1 = recognizable cartilage plaque; 2 = 20% cartilage plaque which was covered up in the longitudinal section of tibia bone; 3 = 50% cartilage plaque which was covered up in the longitudinal section of tibia bone; and 4 = 80% cartilage plaque was covered up in the longitudinal section of tibia bone. The average TD score was calculated for each group by summing the total score for all the chick legs in a group and divided by total number of legs. After that, some tibia bones were fixed in 4% paraformaldehyde and other tibia bones were immediately frozen in liquid nitrogen and stored at −70°C for further analysis.

### 2.3. Hematoxylin & Eosin (H&E) Staining and Immunohistochemistry (IHC)

The fixed (4% paraformaldehyde) tibia bone samples were processed according to Mehmood et al. [30]. The IHC was measured according to previous studies as described by Herzog et al. [31]; the slides were washed in PBS and peroxidase blocking solution (Boster, Wuhan). The slides were incubated with primary antibody against P2RX7 (ABclonal technology, Wuhan, China) with 1 : 2000 dilutions at 4°C overnight. After that they washed with PBS and incubated at 37°C for one and half hour with the horseradish peroxidase-conjugated anti-rabbit secondary antibodies. The immunolabeled slides were observed under the microscope and at last the primary antibodies were removed from negative control.

### 2.4. Quantitative Real-Time Polymerase Chain Reaction (RT-qPCR)

Total RNA of the tibial GP tissues was extracted from each group of chickens using Trizol reagent (Invitrogen, Carlsbad, California, USA), while RNA was transcribed into cDNA according to our previous study [32]. RNA was transcribed into cDNA by using first-strand reverse transcription cDNA kit (Tian Gen, China), according to manufacturer's instructions. Following P2RX7 primer F, ACTTGTGTCCTATGTCGATAAGC and R: AGCAGGTGCGAGGATCATA were used. The RT-qPCR was performed in quadruplex with Step One-Plus™ Real-Time PCR System (Applied Biosystems) for a total volume of 20 *μ*l according to our previous study [32]. All the reaction mixture was normalized against GAPDH gene, while relative quantification of genes was measured using delta Ct (2^−ΔΔCt^) method [33].

### 2.5. Western Blot Analysis

Growth plate of individual tissues was processed according to our previous study [34]. Briefly, after incubating with blocking buffer the membrane was treated with primary antibody (rabbit monoclonal anti-P2RX7, Abcam, 1 : 1000 dilution) at 4°C overnight and then washed 3 times and incubated with secondary antibody. After washing, the images were captured with an imaging system (UVP, Upland, CA, USA).

### 2.6. Statistical Analysis

The results obtained were analyzed by two way ANOVA and student *t*-test to compare the differences between mean values of different groups. The analyses were performed by using SPSS 19.0 software and data were presented as means ± SEM (standard error of means). *P* < 0.05 was used for statistically significant.

## 3. Results

### 3.1. The Mortality Rate Evaluation among Control, TD, and Icariin Groups

**Figure 1:**
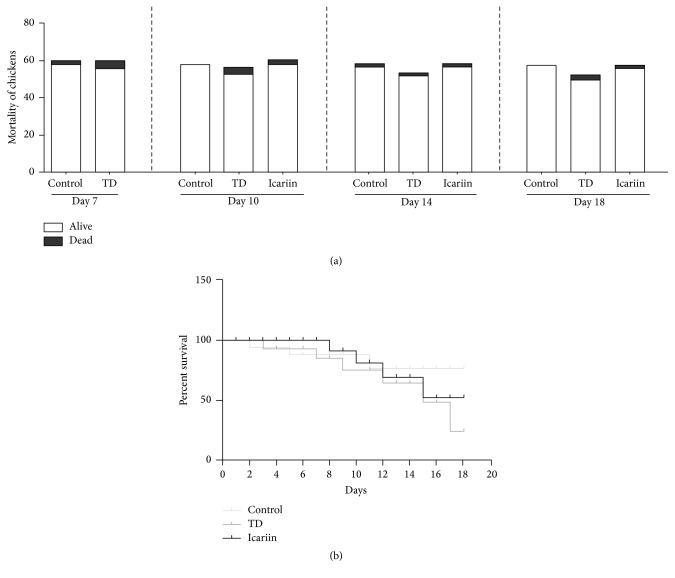
The mortality rate among control, TD, and icariin groups were measured from day 7 to day 18. (a) The mortality of chickens was recorded in days 7, 10, 14, and 18 in different groups. (b) The percent survival was produced with Prism software (GraphPad, USA) from day 1 to day 18 in these three groups.

The mortality was evaluated from day 3 to day 18 in each group. The results showed that mortality rate on day 3 and day 10 was much increased in TD group as compared to control group and then subsequently started to reduce. The mortality rate decreased and TD was restored after removing the thiram from day 14 to day 18. However, in icariin group, the mortality rate of chickens decreased as compared to TD group, as shown in Figure 1.

### 3.2. Icariin Prevented the TD in Chickens

**Figure 2:**
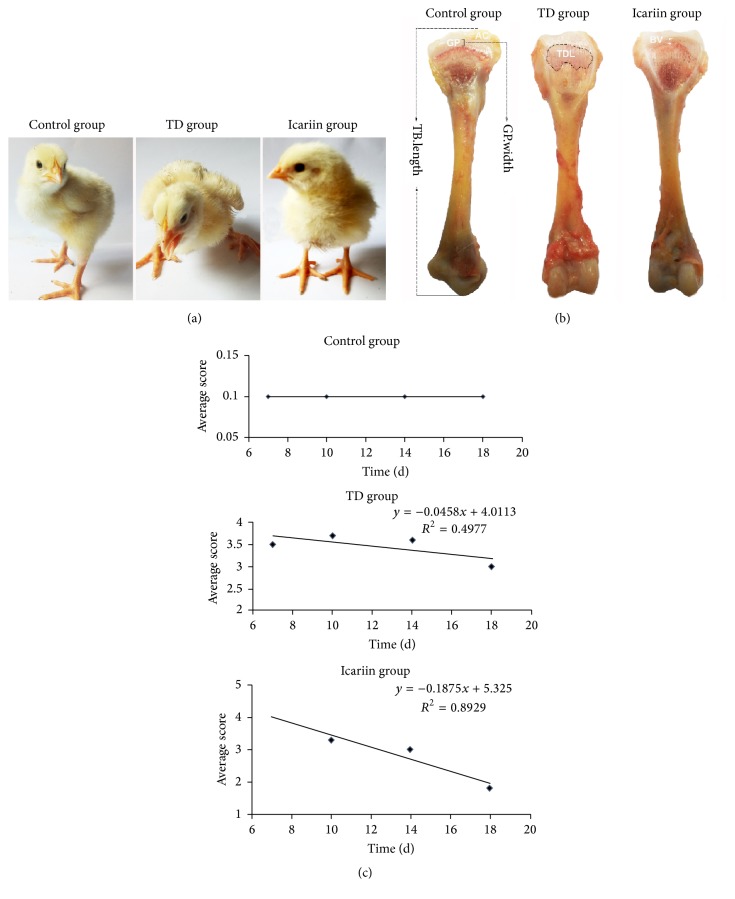
Effects of icariin on thiram induced tibial dyschondroplasia chickens. (a) Lameness was significant in TD groups during the experiment period while no lameness was observed in control group. The icariin group chickens started to regain their ability to walk and stand properly. (b) The morphological changes of proximal tibial growth plates. AC = articular cartilage; GP = growth plate; TDL = tibial dyschondroplasia lesion; BV = blood vessels; TB length = tibia length; GP width = growth plate width. (c) indicates the evaluation of correlation of average score in different groups from day 7 to day 18.

The visual examination indicated the depression and poor body condition in thiram induced TD chickens along with weakness, lameness, and feeding difficulties. As compared with the TD affected chickens, the icariin treatment group regained their ability to stand and walk properly (Figure 2(a)). The continuous administration of icariin in drinking water caused a further decrease in the signs of lameness, and almost all birds reverted to the control level. The morphometry of tibia bone indicated that icariin administration resulted in a significant reduction in growth plate width as compared to thiram fed TD group (Figure 2(b)). The correlation of average score showed that the icariin administration reduced the average TD score in chickens.

### 3.3. Icariin Promoted the Vascularization and Inhibited the Formation of TD Lesion

**Figure 3:**
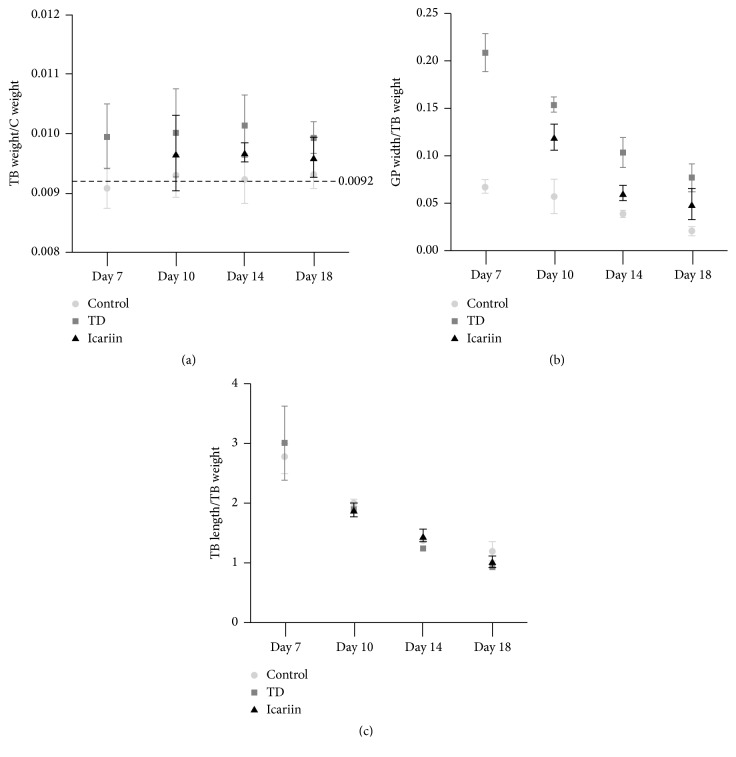
Icariin promoted the vascularization and inhibited the formation of TD lesion in thiram induced tibial dyschondroplasia chickens. (a) The average value of TB weight/C weight was 0.0092 in control group, while it was higher in TD group as compared to control group. However, with the using of icariin, the value becomes lower. (b) The GP width/TB weight was less in control group as compared with TD group, while the icariin treatment was near the control group. (c) The TB length/TB weight was less from day 7 to day 18 with no significant change among three groups. TB weight = tibia weight; C weight = chicken weight; GP width = growth plate width; TB length = tibia length.

To check whether the icariin can promote the vascularization in GP and inhibit the formation of TD lesion, we analyzed the overall tibia bone parameters. The results of tibial parameters revealed that the average value of tibia (TB) weight/chicken (C) weight was 0.0092 in control group, while it was higher in TD group as compared with control group. However, with the usage of icariin, the value became lower (Figure 3(a)). The TB width/TB weight of GP was down in control group as compared with TD group, while icariin treatment group showed very near value to the control group (Figure 3(b)). The TB length/TB weight was decreased from day 7 to day 18 with no significant changes among all the three groups (Figure 3(c)).

### 3.4. TD Score Assay

**Figure 4:**
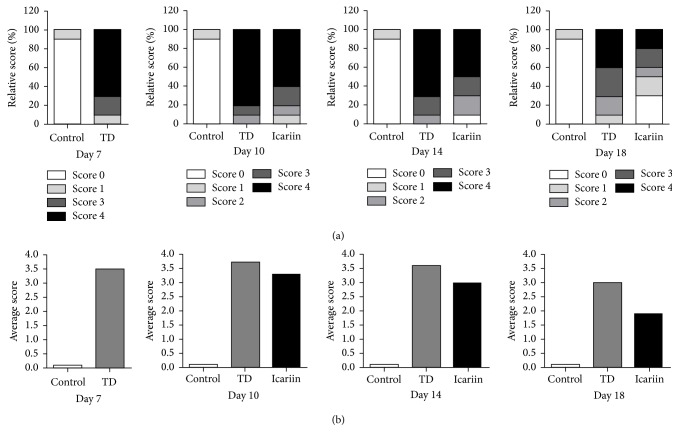
Effect of thiram on tibial dyschondroplasia incidence and treatment of icariin in chickens. All tibial growth plates were dissected to score for TD severity on days 7, 10, 14, and 18 for average and relative TD score. Compared with TD group, the icariin gave significant effect especially on day 14 and day 18.

Thiram induced TD in almost 90% chickens in TD group and tibia showed severe TD lesions (scores 3 and 4), while average TD score was higher compared to control group. In control group, tibia was almost normal phenotypically and average TD score was also less. However, icariin group showed that chicken had restored the TD pathogenesis and more than 50% chickens were found healthy. The average TD score significantly decreased (<2.0) in icariin group on day 18 (Figure 4).

### 3.5. Histological Examination of Tibial Growth Plates

**Figure 5:**
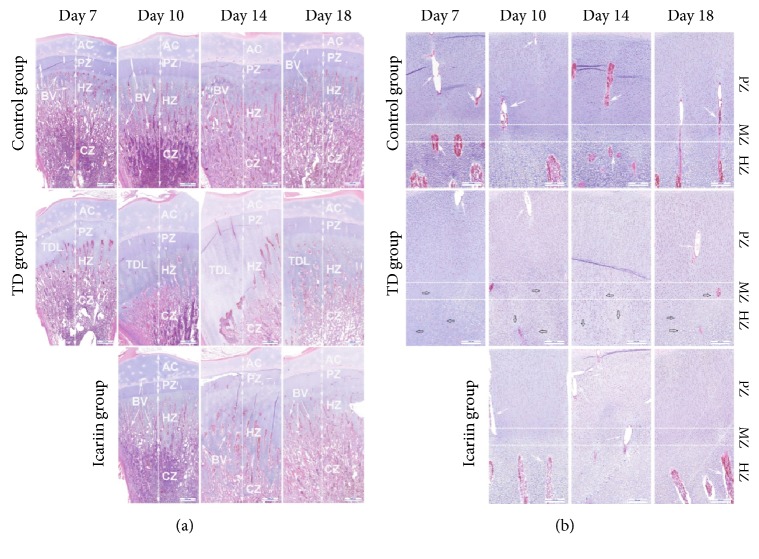
H&E stained histopathology micrograph depicts blood version and cell morphology in tibia bone. The normal group has regular columns of cells surrounded by blood vessels. TD group has necrosis along with less number of blood vessels and avascularized GP. Restored angiogenesis in proliferative zone (PZ) and hypertrophic zone (HZ) of growth plate found with the use of icariin. Arrow indicates blood vessels (BV); articular cartilage (AC); calcified zone (CZ); and TD lesion (TDL).

In order to observe the histopathology micrograph of blood version and cell morphology in tibia bone, H&E staining was performed in control group, TD group, and icariin treatment group. The results showed that the normal GP exhibits regular columns and cells surrounded by large number of blood vessels in proliferative and hypertrophic zone (Figure 5(a)). However, in TD group, a large number of cell death, empty cartilages, disordered arrangements of chondrocytes, and degradation were found in proliferative and hypertrophic zones of growth plate. Calcified area of the GP was shrinked with vascular banding and hypertrophy of cartilage cell. In addition, the vascular necrosis and bone resorption were slow in TD afflicted group (Figure 5(b)). However, icariin restored angiogenesis in the proliferative and hypertrophic zones of growth plate (Figure 5).

### 3.6. Icariin Promoted the Trabecular Bone and Blood Version Formation in TD Affected Chickens

**Figure 6:**
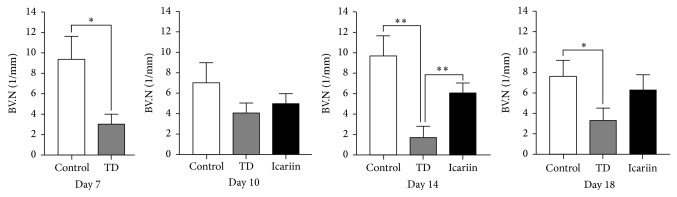
Quantification of blood version numbers assay (BV.N) in different groups from day 7 to day 18. The BV.N was analyzed with Image-Pro Plus. ^*∗*^*P* < 0.05; ^*∗∗*^*P* < 0.01.

**Figure 7:**
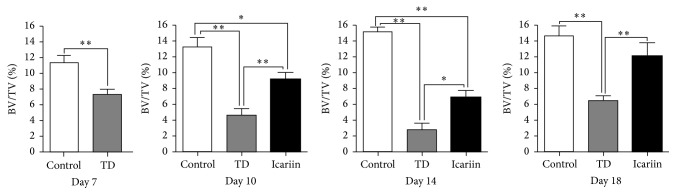
Trabecular bone volume assay of different groups from day 7 to day 18. The BV/TV was analyzed with Image-Pro Plus. ^*∗*^*P* < 0.05; ^*∗∗*^*P* < 0.01.

The results of H&E staining revealed that TD affected chickens had decreased blood version numbers assay (BV.N) and trabecular bone volume (BV/TV) in tibia. However, increased blood version numbers assay (Figure 6) and trabecular bone volume (Figure 7) were found in tibia after icariin administration.

### 3.7. Immunohistochemical Localization of P2RX7 in Tibial Hypertrophic Zone

**Figure 8:**
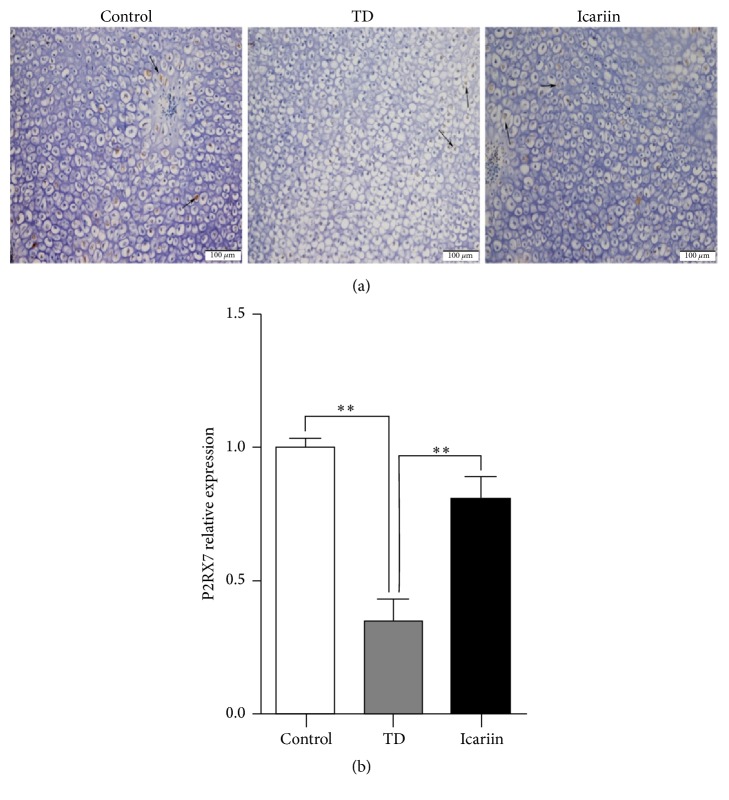
Immunohistochemical localization of P2RX7 in control, TD, and icariin treated chickens in tibial hypertrophic zone. A fewer numbers of cells express P2RX7 in TD-effected chickens relative to control group, and birds returned to normal levels on icariin treatment. Arrow indicates P2RX7 density in brown color. The results were analyzed by Image-Pro® Plus 6.0. ^*∗∗*^*P* < 0.01.

The expression of P2RX7 antibody in the tibia hypertrophic zone was evaluated via immunohistochemistry. The results showed that few numbers of cells were expressed in P2RX7 TD-effected chickens when compared with the control and icariin groups (Figure 8).

### 3.8. The P2RX7 Gene Level and Protein Expression in Growth Plates

**Figure 9:**
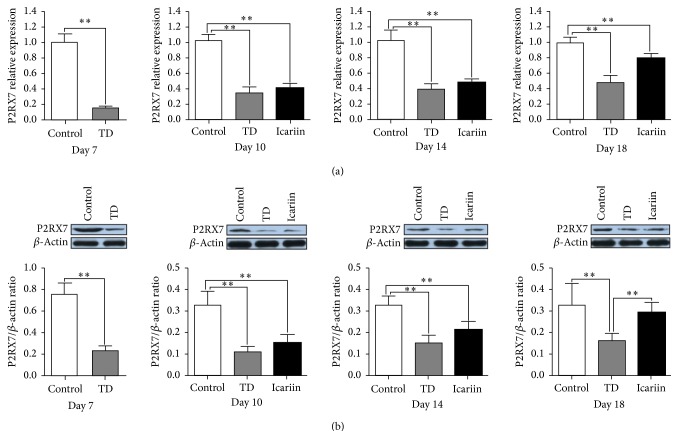
Real-time quantitative PCR analysis and protein levels of P2RX7 in various groups on days 7, 10, 14, and 18. (a) The mRNA level of P2RX7 was confirmed by RT-qPCR. (b) The protein level of P2RX7 was detected by western blotting; the GAPDH and *β*-actin were used as a control in RT-qPCR and western blotting analysis, respectively. Data expressed in arbitrary units as the means ± SEM. ^*∗∗*^*P* < 0.01.

The expression profile of P2RX7 gene involved in the growth plates of chicken indicated that the mRNA expression of P2RX7 was significantly decreased (*P* < 0.05) in TD group as compared to control group. However, icariin administration significantly upregulated the P2RX7 gene expression from day 10 to day 18 (Figure 9(a)). The protein level was assessed by western blotting analysis in tibia bone on days 7, 10, 14, and 18. Results revealed that P2RX7 expression was significantly downregulated (*P* < 0.05) in TD group on days 7, 10, 14, and 18 as compared to control group (*P* < 0.05). But, the level of P2RX7 protein was obviously increased with the administration of icariin on day 14 and day 18 (Figure 9(b)).

## 4. Discussion

Icariin has been widely reported as a potential osteogenic compound for bone repair because it can promote the osteoblast proliferation and osteogenic differentiation [35, 36]. Previous studies have shown that icariin can induce osteogenic differentiation and can protect against osteolysis and inflammation. Therefore, it is widely used in the treatment of bone diseases [37–39]. In this study, we have used the icariin for the treatment of TD, in order to find a new target medicine for the prevention of TD in broiler chickens. Normal growth plate development entails cartilage vascularization and mineralization followed by osteogenesis. But tibial dyschondroplasia is a bone abnormality and characterized by endochondral ossification suffocate, tibial metaphyseal cartilage cell proliferation, avascular, nonmineralized, and white cartilaginous opaque mass in GP [4, 39]. Previous studies show that clinical features of TD include gait abnormality, inability to stand and walk properly, and sometime even death [3, 39, 40]. Our research confirmed the findings of previous reports, like reducing body weight gain and increasing mortality in TD affected chickens. However, after administering the icariin, the chickens become healthy and mortality was reduced significantly.

In order to further study the effect of icariin on pathological changes in TD affected chickens. Our study showed that bone development in chickens starts from immature cartilage cells of static zoon, then proliferative zone, hypertrophy stage and gradually mature, angiogenesis, calcification, and finally bone tissue in proximal growth plate. The vascularization is rich in cartilage zone and cartilage cells are clear with nucleus in the centre. The cell volume is similar to less intercellular substance, and trabecular bone is in mature chondrocytes cartilage capsule during normal growth of chicken. Meanwhile, the excessive growth was found in immature cartilage cells arranged tightly; some nucleus pycnosis and apoptosis were found in severe TD lesions. However, after the treatment of icariin, the histopathology of bone in TD affected chickens approach the normal chickens. Huang et al. [41] stated that hypoxia can enhance the vascularization in the proximal growth plate for bone development. Wang et al. [42] studied that chondrogenesis is regulated by many factors while hypoxia is main factor for the development and regeneration of cartilage because it regulates chondrocytes cell proliferation. Icariin can enhance hypoxia reactive element luciferase activity and play important role in chondrogenesis [42]. Previous studies have shown that the TD affected GP which contains minerals including Na, Mg, P, Cl, S, and Ca just like normal GP and undergoes cell proliferation at the same rate [43, 44]. Moreover, metabolic acidosis, impairment of vascularization, and growth rate have been reported to influence the incidence of TD [10, 45, 46]. However, recently research on TD has found that TD is related to the bone remodeling and reformation.

The P2RX7 is demonstrated to be expressed by major bone cell types, including osteoblasts [46, 47], osteoclasts [48, 49], and osteocytes [50] and the overall effect of a functional P2RX7 on bone metabolism [16]. Some studies also showed that activation of the P2RX7 could inhibit resorption of bone and can stimulate the differentiation of osteoblasts with enhancement of mineralization [51]. Furthermore, P2RX7 plays an essential role in calcium signaling from osteoblasts to osteoclasts in response to mechanical stimulation [52]. In our study, we found significantly decreased level of P2RX7 in TD chickens as compared with normal on day 7 to day 18. Whereas, with the use of icariin, the expression of P2RX7 gene becomes upregulated as compared with TD chickens, the macroscopic observation and pathology examination also confirmed our results.

Chinese traditional medicine and natural products have paid attention to the general public. Present study has found that icariin can activate the P2RX7, which inhibit the resorption of the bone and stimulate the differentiation of osteoblasts, along with the enhancement of mineralization in GP to prevent the TD incidence and its characteristics in broiler chickens. This is the first study that explains the effect of icariin in tibial dyschondroplasia incidence, tibial angiogenesis, and its characteristics via regulating the P2RX7 in chickens.
